# MR Imaging of Intra- and Periarticular Cyst-Like Lesions of the Knee Joint in Workers with Occupational Kneeling

**DOI:** 10.1155/2012/843970

**Published:** 2012-04-14

**Authors:** Søren Rytter, Lilli Kirkeskov Jensen, Jens Peter Bonde, Niels Egund

**Affiliations:** ^1^Department of Orthopaedic Surgery, Hospital Unit West, Lægårdvej 12, 7500 Holstebro, Denmark; ^2^Department of Environmental and Occupational Medicine, Bispebjerg Hospital, Bispebjerg Bakke 23, 2400 Copenhagen NV, Denmark; ^3^Department of Radiology, Aarhus University Hospital, Nørrebrogade 44, 8000 Aarhus C, Denmark

## Abstract

*Objective*. To determine the risk of intra- and periarticular cyst-like lesions of the knee joint in occupational kneeling. *Methods*. Magnetic resonance imaging of both knees (*n* = 282) was conducted in 92 male floor layers and 49 male graphic designers (referents), with a mean age of 55.6 years (range 42–70 years). The prevalence of cyst-like lesions was computed among floor layers and graphic designers, respectively, and associations with occupation summarized by odds ratio (OR) with 95% confidence intervals (CIs). Using logistic regression, models were adjusted for age, body mass index, knee injuries, and knee-straining sports. *Results*. Floor layers had a significantly higher prevalence of cyst-like lesions in the posterior part of the knee joint compared to graphic designers (OR 2.70, 95% CI 1.50–4.84). Floor layers also had a higher prevalence of fluid collections in the popliteus tendon recess (OR 2.17, 95% CI 0.99–4.77) and large cystic lesions of the popliteus muscle (OR 3.83, 95% CI 0.78–18.89). The prevalence of cystic lesions in the anterior part of the knee joint was low among floor layers (8.7%) and there was no significant difference between the two trade groups (*P* = 0.34). *Conclusions*. Occupational kneeling increases the risk of cyst-like lesions in the posterior part of the knee joint.

## 1. Introduction

Benign cystic lesions of the knee region in the form of fluid collections in synovial recesses, bursae, and ganglion cysts are common and often discovered incidentally in routine magnetic resonance imaging (MRI) examinations. MR has proved to be superior compared to other imaging techniques in the detection of cystic lesions of the knee [1, 2] and add information of the anatomical relationship with adjacent structures and concurrent knee pathologies [3]. The prevalence of cystic lesions has been described in both symptomatic and asymptomatic knees in previous studies [4–6] but only a few studies have examined the association between occupation-related kneeling and squatting and knee pathologies demonstrated by MRI. Based on MRI, Amin et al. [7] showed that both heavy lifting and frequent kneeling and squatting increased the risk for knee cartilage degeneration, particularly at the patellofemoral (PF) joint, and we have in a previous study showed that occupational kneeling increases the risk of degenerative tears in the medial meniscus [8]. However, to our knowledge, no previous MRI studies have examined the relationship between occupational kneeling and the prevalence of intra- and periarticular cyst-like lesions of the knee joint. Biomechanical studies have shown that tibiofemoral (TF) contact forces increase considerably during deep knee flexion and force joint fluid into the posterior part of the knee joint [9–12]. Direct and indirect loading of the knee joint during kneeling work demands could, therefore, be a predisposing factor in the development of both intra- and periarticular disorders of the knee joint. Many workers in the construction industry are exposed to knee-straining work activities but floor layers are particularly exposed. Studies have shown that floor layers on average spend 40–50% of the daily work time in kneeling work positions and that floor layers have a significantly increased prevalence of knee complaints [13–16]. However, the high prevalence of knee complaints may not be explained by knee osteoarthritis (OA) and meniscal tears alone but could be attributable to other knee pathologies, for example, intra- and periarticular cystic-like lesions.

The aim of this study was to examine the occurrence of MRI-detected intra- and periarticular cyst-like lesions including bursitides of the knee joint in floor layers in comparison with a group of graphic designers without any occupationally related knee demands. We, furthermore, evaluated the association between MRI findings and self-reported knee complaints and assessed the association of concomitant joint effusion, and cyst-like lesions potentially communicating with the knee joint, meniscal tears, and knee OA.

## 2. Materials and Methods

### 2.1. Study Sample

A Danish sample of 286 male floor layers and 370 male graphic designers was established from trade union rosters comprising members aged 36–70 years in 2004 and with residence in Copenhagen or Aarhus, Denmark. Floor layers install different kinds of floorings and their work tasks involve prolonged kneeling and frequent change from kneeling to standing work positions. Graphic designers constituted the reference group. They work primarily with the layout of texts and advertisements using visual display units and their work did not include any knee demands.

A self-administered questionnaire was forwarded to the study sample. Respondents constituted 253 (89%) floor layers and 290 (78%) graphic designers. The questionnaire provided data on anthropometrical characteristics, history of employment, knee complaints (ache, pain, or nuisance during the past 12 mo), knee injuries (fractures involving the knee, meniscal injuries, anterior (ACL), or posterior (PCL) cruciate ligament lesions), and knee-straining sports activity defined as ever participated in football, handball, badminton, tennis, volleyball, ice hockey, or weight lifting. Respondents were invited to participate in additional examinations. Written informed consent was obtained from 156 floor layers and 152 graphic designers. Among those, a random sample of 92 (Copenhagen, *n* = 45; Aarhus, *n* = 47) floor layers and 49 graphic designers (Copenhagen) underwent an MRI examination of both knees (total 282 knees). Examinations were conducted at 2 MR centres in Aarhus (Centre I) and Copenhagen (Centre II), respectively. The Central Danish Region Committees on Biomedical Research Ethics approved the study protocol.

### 2.2. MRI Acquisition

MRI was performed by a 1.5 Tesla scanner (SymphonyVision, Siemens Medical Systems, Erlangen, Germany) at Centre I and by a 1.5 Tesla scanner (Infinion, Philips, Best, The Netherlands) at Centre II. The following MRI sequences was obtained at Centre I: sagittal proton density fat-saturated turbo spin echo (TR/TE, 3300/15 ms) and sagittal and coronal T2-weighted (4000/86 ms) fat-saturated turbo spin-echo; coronal T1-weighted (608/20 ms) spin-echo sequences and axial proton density fat-saturated turbo spin echo (3450/15 ms). The section thickness was 4 mm with an intersection gap of 0.4 mm; field of view was 200 × 200 mm and matrix 512 in all sequences. At Centre II, the MRI sequences included sagittal proton density fat-saturated turbo spin echo (TR/TE, 2500/18 ms) and sagittal and coronal T2-weighted (4000/85 ms) fat-saturated turbo spin-echo; coronal T1-weighted (400/13 ms) spin-echo and axial proton density fat-saturated turbo spin echo (2880/17 ms). The section thickness was 4 mm with an intersection gap of 0.4 mm; field of view was 150 × 150 mm and matrix 512 in all sequences.

### 2.3. MRI Assessment

The same experienced musculoskeletal radiologist (NE) evaluated each of the 282 MRI examinations. The radiologist was blinded to any medical history of knee disorders among participants. Due to differences in the appearance of MRI at the two centres, blinding of occupational affiliation was incomplete for participants from Centre I, who were all floor layers. Blinding of occupational affiliation was complete for all participants from Centre II.

**Figure 1:**
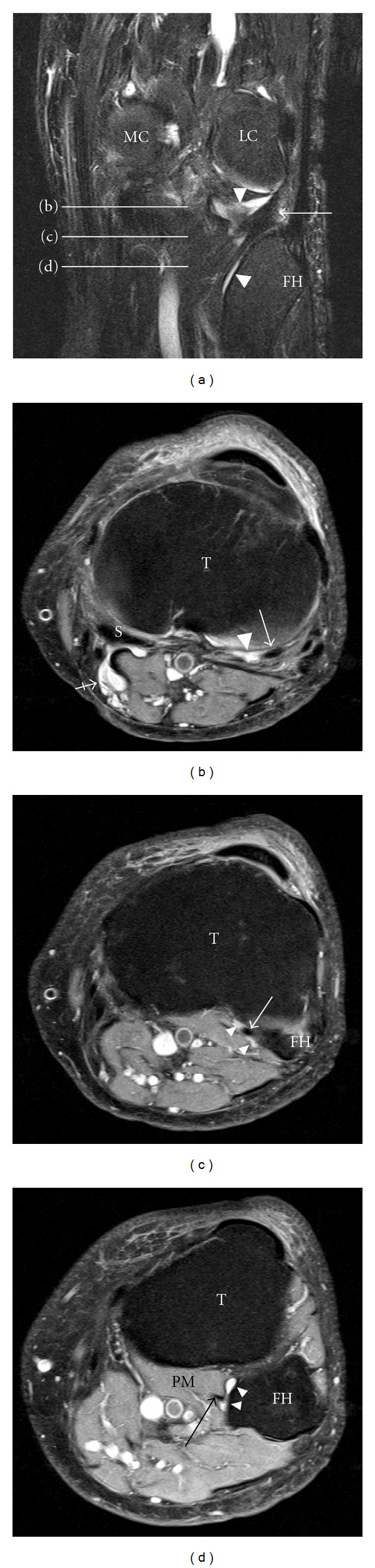
Synovial recess along the popliteus tendon presented at three distances from the popliteus hiatus. (a) Overview of the course of the popliteus tendon (arrow) and its intimately related synovial recess (white arrow heads) on a coronal MRI fat saturated T2-weighted image of the dorsal aspect of the left knee without effusion in a 64-year-old male floor layer. The white lines illustrate the level of the axial proton density fat-saturated images b, c, and d. (b) The popliteus tendon (arrow) is embedded in the synovial recess (white arrow head) close to the joint space of the knee. Popliteal cyst (crossed arrow) communicates with a subgastrocnemius bursa (black arrowhead). (c, d) Note the characteristic appearance of the not distended synovial recess (arrow head) of a branch of the popliteus tendon (arrow) at the level of the fibular head (FH). The recess should not be mistaken for a ganglion cyst originating from the proximal tibiofibular joint. MC and LC, medial and lateral femoral condyle; T, tibia; S, semimembranosus tendon; PM, popliteus muscle.

**Figure 2:**
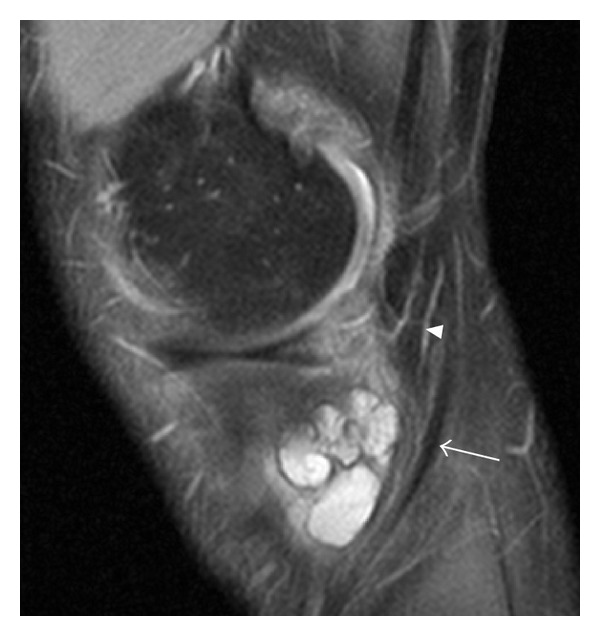
Pes anserine bursitis. Sagittal proton density fat-saturated image of the medial aspect of the left knee in a 47-year-old male floor layer. Arrow, semitendinosus tendon; arrow head, semimembranosus tendon.

**Figure 3:**
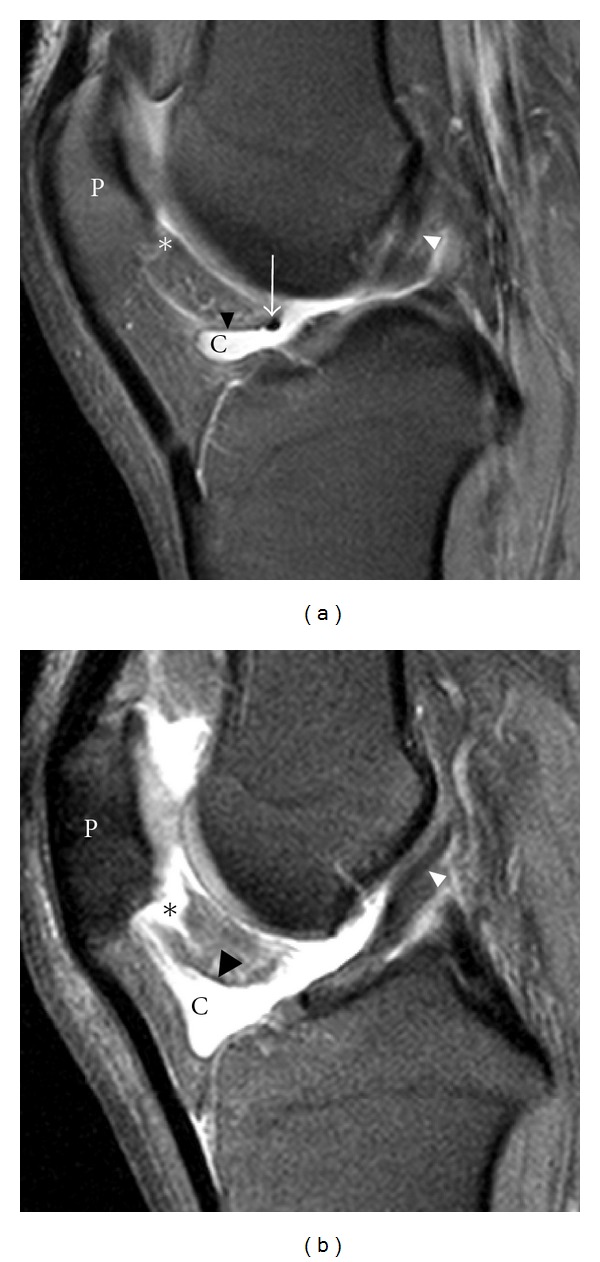
Fluid filled synovial clefts of Hoffa's fat pad in the knees of 2 floor layers. In these sagittal proton density fat-saturated images of the right knee in a 43-year-old male floor layers with mild (a) and a 50-year-old male floor layer with moderate (b) effusion of the inferior, horizontal Hoffa's cleft there is a communication with the superior cleft (*) along the ligamentum mucosum (black arrow head). Arrow, anterior transverse ligament; white arrow head, anterior cruciate ligament; P, patella.

**Figure 4:**
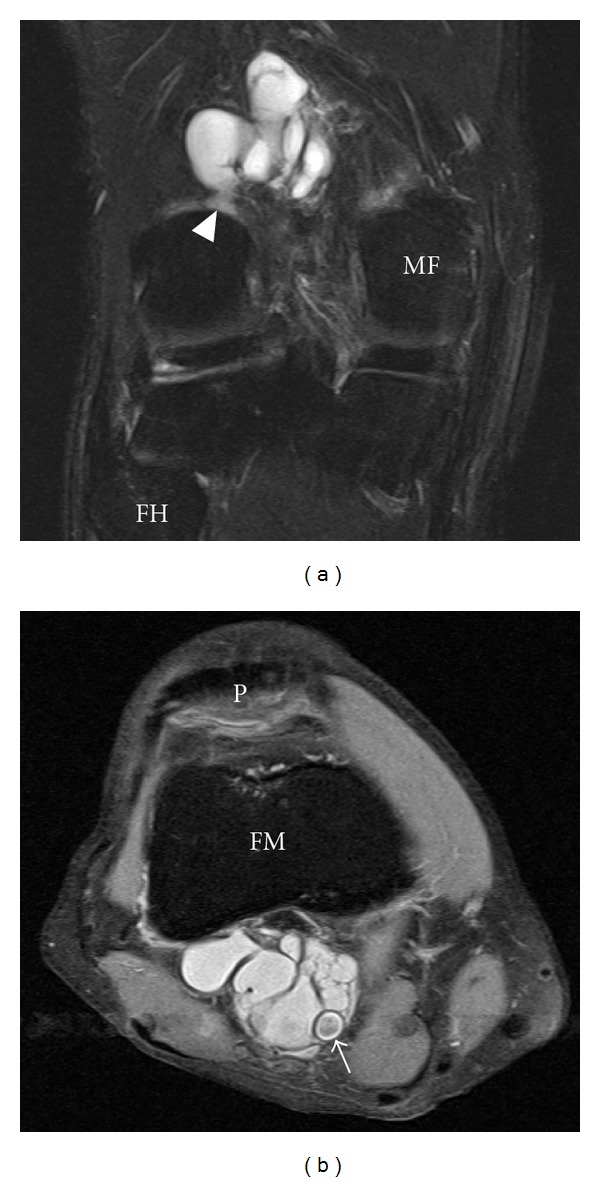
Cluster of communicating extra capsular synovial cysts at the dorsal aspect of the femoral metaphysis. (a) Coronal fat-saturated T2 and (b) axial proton density fat-saturated MR images of the right knee without effusion in a 51-year-old male floor layer. The lesion takes it origin from the intracapsular synovial recess at the dorsal cranial aspect of the lateral femoral condyle through a defect in the capsule (arrow head). The majority of these lesions detected in floor layers originated similarly but from the medial femoral condyle. FH, fibular head; MF, medial femoral condyle; FM, femoral metaphysis; P, patella; arrow, popliteal artery.

The presence of fluid accumulations of the following bursae and synovial recesses were evaluated: semimembranosus-gastrocnemius (Popliteal cyst—Figure 1), medial and lateral subgastrocnemius [4], prepatellar, superficial, and deep infrapatellar, medial (MCL), and lateral (LCL) collateral ligament, biceps femoris, iliotibial, anserine, and semimembranosus-gracilis bursitides (Figure 2) and Hoffa's fat pad recesses (Figure 3), [1–4, 17, 18]. Other cyst-like lesions included meniscal cysts, ganglion cysts of the cruciate ligaments, and extracapsular synovial cysts (Figure 4). Two types of fluid collections along the popliteus tendon (PT) were distinguished: cystic lesions originating from the proximal tibiofibular joint [19] and the PT recesses [1]. Insertional cysts [17, 20] were assessed at the cruciate and collateral ligaments. The presence or absence (0 = none, 1 = present) of cystic lesions was registered and the length of fluid collections in the PT recess was measured in mm from the upper demarcation line of the proximal tibiofibular joint and distally. Knee joint effusion was registered (0 = none, 1 = present) as the presence of a localized fluid collection in the intercondylar area, in the medial or lateral recess, or as a generalized fluid collection that distended the suprapatellar recess [21]. Abnormalities in the MR signal intensities predicting meniscal tears were classified in grades 1–3 [8] and radiographic knee OA were classified using a modified Ahlbäck scale of joint space narrowing (JSN) [22].

### 2.4. Statistical Analysis

The prevalence of cyst-like lesions was computed among floor layers and graphic designers, respectively, and associations with occupation assessed by logistic regression and summarized by odds ratios (OR) with 95% confidence intervals (CIs). Models were adjusted for age, body mass index (BMI), knee injuries, and knee-straining sports. Using logistic regression, we additionally assessed the association between cyst-like lesions and knee complaints and furthermore associations between joint effusion and the presence of cystic lesions potentially communicating with the knee joint capsule, meniscal lesions (grade 3) and radiographic knee OA (JSN ≥ 25%). The adjusted OR with 95% CI was computed, and independent variables incorporated in the model of adjusted results were occupation, age, BMI, knee injuries, and knee-straining sports. The observational unit in the statistical analyses was at the subject level.

Statistical analyses were performed using Stata (StataCorp LP, College Station, TX, USA).

## 3. Results

Characteristics of the study sample are given in Table 1. The age of participants ranged from 42 to 70 years. Mean age and trade seniority were higher among graphic designers compared to floor layers, and graphic designers also had a higher proportion of previous knee injuries and knee-straining sports experience than floor layers. The two study groups were comparable regarding average BMI and the proportion of knee complaints.

**Figure 5:**
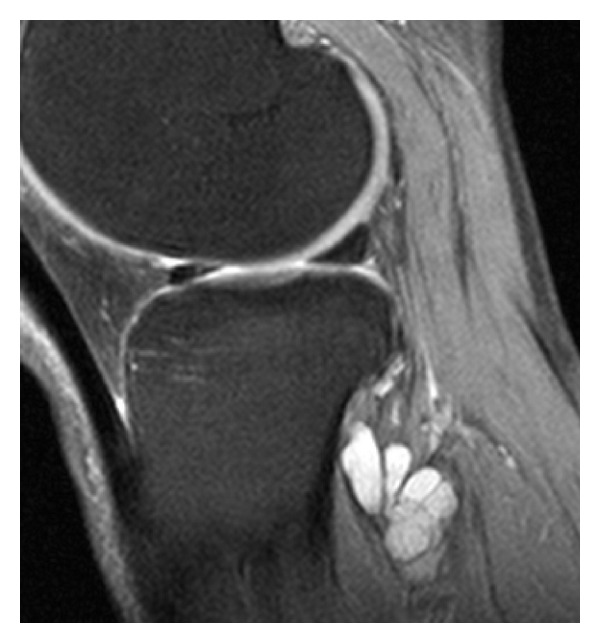
Multiple cysts within the popliteus muscle. Sagittal proton fat-saturated MR image of the right knee without effusion or synovitis in a 56-year-old male floor layer. The lesions represent an abnormal extension of the popliteus tendon synovial recess.

Fluid accumulations in various bursae were a common finding in both trade groups (Table 2(a)). Among 141 participants, 113 (80.1%) had MRI findings consistent with bursal fluid collections around the knee joint. Comparing the two trade groups, 85.8% of the floor layers were classified as having at least one bursal fluid collection in one or both knees compared to 69.3% among graphic designers (OR 2.94, 95% CI 1.17–7.44). The prevalence differed in various sites of the knee joint and was generally higher among floor layers than graphic designers except for fluid collection in the prepatellar bursa. The prevalence of other fluid collections is given in Table 2(b). Floor layers had a higher prevalence of fluid collections in the PT recess (Figure 1) compared to graphic designers, and the average extents of PT recesses were also larger in floor layers (mean 23.7 mm, 95% CI 21.3–26.1) than in graphic designers (mean 20.1 mm, 95% CI 17.6–22.6; **P** < 0.05). An unexpected finding was cystic lesions within the lateral portion of the popliteus muscle (Figure 5). These abnormalities were seen with and without communication to a not distended PT recess and occurred almost exclusively in floor layers. Cystic lesions originating from the proximal tibiofibular joint were not detected. Extracapsular synovial cysts extending in the proximal direction from capsular defects of the dorsal aspect of the femoral condyles (Figure 4) and fluid collections in the MCL, LCL, biceps femoris, and anserine-semimembranosus-gracilis bursae (Figure 2) were only registered in floor layers. Although nonsignificant, floor layers also had a higher prevalence of medial parameniscal cysts and fluid-filled recesses in the Hoffa fat pad (Figure 3).

The prevalence of cystic lesions in the anterior part of the knee (prepatellar, superficial and deep infrapatellar, and anserine bursae) was found in 16 of 184 (8.7%) knees in floor layers compared to 12 of 98 (12.2%) knees in graphic designers (OR 0.68, 95% CI 0.29–1.66). Cystic lesions in the posterior part of the knee; semimembranosus-gastrocnemius (Figure 1) and subgastrocnemius bursitides (medial and/or lateral), and extracapsular synovial cysts (Figure 4), were a common finding but significantly more prevalent in floor layers and found in 149 of 184 (80.9%) knees compared to 60 of 98 (61.2%) knees in graphic designers (OR 2.70, 95% CI 1.50–4.84).

The association between knee joint effusions and cyst-like lesions potentially communicating with the knee joint capsule, meniscal tears, and radiographic knee OA are given in Table 3. The prevalence of joint effusions was 36.9% (34/92) and 28.5% (14/49) in floor layers and graphic designers (**P** = 0.32), respectively. Although nonsignificant, joint effusions were more frequently associated with fluid collections in the semimembranosus-gastrocnemius and the subgastrocnemius bursae than PT recesses and extracapsular synovial cysts. However, a stronger association was found with meniscal tears, and knee OA.

Knee complaints were most frequently associated with ACL insertional cysts, and more common among subjects with fluid collections in the periarticular bursae than subjects with fluid collections in the peripatellar bursae. The risk of knee complaints was low among subjects with cystic lesions around the medial (parameniscal cysts) and lateral (PT recesses) meniscus (Table 4).

## 4. Discussion

In a sample of floor layers exposed to occupational kneeling, we found a significantly higher prevalence of cyst-like lesions in the posterior part of the knee joint compared to a reference group of graphic designers and our findings revealed surprisingly few floor layers with cystic lesions in the anterior part of the knee joint.

Fluid collections in the subgastrocnemius bursa were the most prevalent finding in our material. The subgastrocnemius bursa is located above the knee joint and deep in relation to the medial gastrocnemius muscle where it communicates with the posterior joint capsule. It has been shown that this bursa commonly communicates with the semimembranosus-gastrocnemius bursa. In the present study, 66.7% (40/60) of those with a fluid collection in the semimembranosus-gastrocnemius bursa had a simultaneous collection in the subgastrocnemius bursa (**P** = 0.05). This supports the theory of communication between the two bursae.

The prevalence of fluid collections in the semimembranosus-gastrocnemius (Popliteal cyst) bursa on MRI reported in the literature ranges from 5% to 38% [3]. Our results seem to be in agreement with these results, although we observed a higher prevalence among floor layers (46.7%) compared to graphic designers (34.7%). The pathogenesis of this disorder has been explained by a slit-like communication of the posterior joint capsule and the bursae. Compared to the posterolateral corner of the knee joint that obtains reinforcement from the ligament of Wrisberg, the PT, and the PCL, the posteromedial corner has only supplemental reinforcement from the posterior horn of the medial meniscus [23]. This relative weakness of the posteromedial joint capsule may allow joint fluid to move from the knee joint into the bursa. In addition, studies have shown that pull from the semimembranosus tendon and the medial meniscus during knee flexion opens this slit-like communication [23]. However, cadaveric studies have shown that roughly 50% of popliteal cysts do not communicate with the knee joint [24, 25]. Other mechanisms may, therefore, be involved in the pathogenesis. Bursal enlargement could be the result of multiple microtrauma of the bursa related to muscle contraction during repeated kneeling [25]. Another hypothesis is that kneeling, which has been shown to increase contact stress in the medial TF compartment significantly, may predispose to progressive thinning of the posteromedial joint capsule due to tearing forces emanating from the pull of the meniscus and the semimembranosus tendon [10, 24]. Work tasks with repeated and prolonged kneeling could, therefore, explain the high prevalence of fluid collections in the semimembranosus-gastrocnemius bursa found in floor layers.

Excess fluid accumulation in the prepatellar bursa has traditionally been associated with direct external pressure due to repeated kneeling [13, 26–28]. Although few cases, we found a higher prevalence of excess fluid accumulation in the infrapatellar bursae compared to the prepatellar in floor layers. Many of the floor layers' work tasks are performed in knee angles above 90-degree flexion. In these work positions, most of the directly related stresses between the knee and underlay are located around the tibiae tubercle, and not between the patellae and the underlay. Mechanical stress in this area could explain the higher prevalence of excess fluid accumulation in the infrapatellar and anserine bursae (Figure 2) in floor layers. Our results are in accordance with a previous ultrasound study by Myllymäki et al. [28] that found a higher prevalence of fluid accumulation in the infrapatellar bursa compared to the prepatellar bursa among carpet-layers compared to a reference group of painters.

Ganglion cysts originating from the tibiofibular joint [19] were not detected, but fluid collections along the PT recesses [1] were a common finding and showed a higher prevalence in floor layers compared to graphic designers; significantly more floor layers had lesions in both knees. Apparently, there is some confusion in the literature distinguishing ganglion cysts originating from the proximal tibiofibular joint from PT recesses. These ganglion cysts have a characteristic anatomical location and MR appearance [2] and are rare with a prevalence of 0.76% [19]. Also, PT recesses have characteristic MR appearances on axial and coronal MR slices (Figure 1), [1]. However, these recesses have in many publications been interpreted on sagittal MR images to originate from the proximal tibiofibular joint [3, 4, 17, 29–31]. The prevalence of fluid collections along the PT varies between 0% and 7% in asymptomatic knees [4, 29–31] and between 3% and 12% in symptomatic knees [29–31]. The high prevalence of PT synovial recesses in this study sample may be explained by differences in the MR sequences used. The pathogenesis of PT recesses may be comparable to that of popliteal cysts [1]. To our knowledge, the MR appearances of multilobulated cystic lesions in the popliteus muscle (Figure 5) found in 13 knees of floor layers and 2 control knees have not been described previously. In most of the cases, the images of these cystic lesions demonstrated continuity to PT recesses.

Apart from meniscal tears, and knee OA, we did not find significant relations between concomitant knee joint effusion and synovial recesses along the PT or other cyst-like lesions potentially communicating with the knee joint capsule. The presence of cyst-like lesions communicating with the knee joint may, therefore, exist in knees without pathologic joint effusion and not act as a marker of joint effusion, which also has been suggested in previous studies [29, 30].

We found a high prevalence of knee complaints in both study groups but no clear association with MRI findings. The only prevalent finding was a positive association with ACL insertional cysts. These findings are in accordance with results from a neurosensory knee mapping study [32]. By probing the knee joint without anaesthesia, it was shown that the area of cruciate ligaments insertion in particular was associated with pain. However, bursal tissue has a rich neural innervation of free nerve endings, and deformation of bursae may also be responsible for pain associated with bursal fluid accumulation [33]. The association between cyst-like lesions and knee pain has, however, been conflicting in previous studies. Although non-significant, our findings showed a higher association between knee complaints and fluid accumulations in periarticular bursae compared to accumulations in peripatellar bursae. This finding corresponds to earlier results of Hill et al. [31] who in an MRI study found an equal distribution of peripatellar bursitis among subjects with and without knee pain, whereas periarticular bursitis was significantly more prevalent in subjects with knee pain. Guermazi et al. [30] examined the association between intra- and periarticular cyst-like lesions and incident knee pain, and they found no significant associations. They suggested that such lesions may be a secondary phenomenon, which occurs in painful knees rather than a primary trigger of knee pain. Current and previous findings indicate that not all cyst-like lesions may be clinically significant. However, they may in addition to knee OA and meniscal tears to some extent explain previous reports of significantly increased prevalence of knee complaints among floor layers [8, 13, 15, 22].

Some limitations of this study warrant discussion. First, the power of the study was limited due to the small study sample and the low response rate from questionnaires. Second, results could be biased if the decision to participate in the study was differentially influenced by previous or current knee complaints. As discussed in a previous publication with the same study sample, our analysis revealed that graphic designers had a greater tendency to participate if knee symptoms were present [relative risk (RR) 2.7, 95% CI 1.7–4.3], than among floor layers (RR 1.2, 95% CI 0.9–1.6) [8]. This may have introduced bias and led to an underestimation of the difference between the two study groups. Third, differential selection of workers toward different occupations depending on their health status may be inventible in occupations with high physical demands and a healthy-worker selection may have influenced results [34]. However, such selection mechanisms would typically result in an underestimation of the investigated association. Finally, we acknowledge that some of the MR findings may be changes discovered incidentally in routine MR imaging and that we do not know the clinical relevance of these findings. However, our results may be of interest due to the marked difference in the prevalence of findings in the two trade groups as previous studies have shown a high occurrence of unexplained knee complaints among workers exposed to frequent and prolonged kneeling.

In summary, this was the first study to assess the relationship between occupational kneeling and intra- and periarticular cyst-like lesions of the knee joint. Results revealed a significantly higher prevalence of cystic lesions in the posterior part of the knee joint in floor layers compared to a reference group of graphic designers and findings call attention to a possible association with occupational kneeling.

## Figures and Tables

**Table 1:** Characteristics of the study sample, floor layers (*n* = 92), and graphic designers (*n* = 49).

	Floor layers		Graphic designers		*P*-value
Age, yrs (mean, SD)	54.5	7.2	57.7	5.6	*P* < 0.05
Trade seniority*, yrs (mean, SD)	29.6	9.8	35.9	6.5	*P* < 0.05
BMI^†^, kg/m^2^	26.2	3.4	26.6	4.8	*P* = 0.53
Knee complaints^‡^, *n* (%)	46	50.0	24	48.9	*P* = 0.91
Knee injuries^§^, *n* (%)	5	5.4	10	20.4	*P* < 0.05
Knee-straining sports^¶^, (%)	46	50.0	36	73.5	*P* < 0.05

*Duration of employment in the trade.

^†^Body mass index.

^‡^Ache, pain, or nuisance during the past 12 mo.

^§^Fractures of the knee joint, meniscal injuries, cruciate ligament ruptures.

^¶^Football, handball, badminton, tennis, volleyball, ice hockey, and weight lifting.

**Table:** (a)

Bursae	Floor layers		Graphic designers			
	*n *	(%)	*n *	(%)	OR*	95% CI^†^
Subgastrocnemius	57	(61.9)	24	(48.9)	1.76	0.82–3.75
*Unilateral *	30	(52.6)	9	(37.5)	2.81	1.03–7.67
*Bilateral *	27	(47.4)	15	(62.5)	1.25	0.52–2.99

Semimembranosus-gastrocnemius	43	(46.7)	17	(34.7)	1.49	0.67–3.29
*Unilateral *	33	(76.7)	13	(76.5)	1.39	0.58–3.35
*Bilateral *	10	(23.3)	4	(23.5)	1.74	0.46–6.58

Prepatellar	1	(1.1)	5	(10.2)	0.16	0.02–1.55
*Unilateral *	1	(100)	3	(60.0)	0.33	0.03–4.18
*Bilateral *	0	(—)	2	(40.0)	—	—

Superficial infrapatellar	4	(4.3)	2	(4.1)	0.90	0.14–5.75
*Unilateral *	4	(100)	1	(50.0)	1.78	0.17–19.21
*Bilateral *	0	(—)	1	(50.0)	—	—

Deep infrapatellar	10	(10.8)	2	(4.1)	3.53	0.64–19.65
*Unilateral *	9	(90.0)	2	(100)	3.22	0.56–18.32
*Bilateral *	1	(10.0)	0	(—)	—	—

Anserine	5	(5.4)	0	(—)	—	—
*Unilateral *	5	(100)	0	(—)	—	—
*Bilateral *	0	(—)	0	(—)	—	—

Bursae, others^‡^	7	(7.6)	0	(—)	—	—
*Unilateral *	5	(71.4)	0	(—)	—	—
*Bilateral *	2	(28.6)	0	(—)	—	—

**Table:** (b)

Other cyst-like lesions	Floor layers		Graphic designers			
	*n*	(%)	*n*	(%)	OR*	95% CI^†^

Popliteus						
Tendon recesses	53	(51.0)	22	(34.7)	2.17	0.99–4.77
*Unilateral *	24	(57.4)	19	(88.2)	1.14	0.48–2.73
*Bilateral *	29	(42.6)	3	(11.8)	8.89	2.21–35.74
Muscle cysts	13	(14.1)	2	(4.1)	3.83	0.78–18.89
*Unilateral *	12	(92.3)	2	(100)	3.40	0.68–17.06
*Bilateral *	1	(7.7)	0	(—)	—	—

Extracapsular synovial cysts^§^	9	(9.8)	0	(—)	—	—
* Unilateral*	6	(66.7)	0	(—)	—	—
* Bilateral*	3	(33.3)	0	(—)	—	—

Insertional cysts						
ACL^¶^	10	(10.8)	7	(14.3)	1.05	0.33–3.39
*Unilateral *	8	(80.0)	7	(100)	0.91	0.27–3.07
*Bilateral *	2	(20.0)	0	(—)	—	—
PCL^||^	4	(4.3)	0	(—)	—	—
*Unilateral *	3	(75.0)	0	(—)	—	—
*Bilateral *	1	(25.0)	0	(—)	—	—

Hoffa fat pad recesses	18	(18.5)	5	(10.2)	1.80	0.59–5.48
* Unilateral *	11	(58.8)	5	(100)	1.04	0.31–3.44
* Bilateral*	7	(41.2)	0	(—)	—	—

Parameniscal cysts						
Medial	12	(13.0)	2	(4.1)	4.28	0.80–22.87
*Unilateral *	10	(83.3)	2	(100)	3.24	0.59–17.69
*Bilateral *	2	(16.7)	0	(—)	—	—
Lateral	3	(3.2)	2	(4.1)	1.14	0.17–7.87
*Unilateral *	3	(100)	1	(50.0)	2.37	0.21–26.43
* Bilateral *	0	(—)	1	(50.0)	—	—

*Odds ratio calculated relative to graphic designers. Adjusted for age, body mass index, knee injuries, and knee-straining sports.

^†^Confidence interval.

^‡^Medial and lateral collateral ligament, biceps femoris, semimembranosus-gracilis.

^§^From capsular defects of the dorsal femoral condyles.

^¶^Anterior cruciate ligament.

^||^Posterior cruciate ligament.

**Table 3:** Association between knee joint effusion and cyst-like lesions*, meniscal tears, and radiographic tibiofemoral (TF), and patellofemoral (PF) osteoarthritis (OA).

Type of lesion	Floor layers and graphic designers (*n* = 141)			
	*N*	Joint effusion, *n* (%)	OR^†^	95% CI^‡^
Popliteus tendon recesses	75	24 (32.0)	0.85	0.53–1.38
Subgastrocnemius bursae	81	31 (38.3)	1.43	0.93–2.22
Semimembranosus- gastrocnemius bursa	60	22 (36.7)	1.25	0.73–2.12
Extracapsular synovial cysts^§^	9	3 (33.3)	1.20	0.44–3.28
Medial meniscal tears^¶^	88	32 (36.4)	1.36	0.88–2.10
Lateral meniscal tears^¶^	23	12 (52.2)	2.52	1.06–5.96
TF OA^||^	25	14 (56.0)	2.90	1.12–7.54
PF OA^||^	16	9 (56.3)	2.96	0.94–9.33

*Cystic lesions potentially communicating with the knee joint capsule.

^†^Odds ratio calculated relative to joint effusion (*n* = 48) in the entire study sample. Adjusted for occupation, age, body mass index, knee injuries, and knee-straining sports.

^‡^Confidence interval.

^§^From capsular defects of the dorsal femoral condyles.

^¶^Grade 3 [8].

^||^ **J**oint space narrowing ≥25%. Missing radiographs in 2 floor layers and 1 graphic designer [22].

**Table 4:** Association between knee complaints*and cyst-like lesions.

Type of lesion	Floor layers and Graphic designers (*n* = 141)			
	*N*	Knee complaints, *n* (%)	OR^†^	95% CI^‡^
Periarticular bursae^§^	102	53 (52.0)	1.40	0.65–3.04
Peripatellar bursae^¶^	18	8 (44.4)	0.61	0.21–1.78
Joint effusion	48	27 (56.3)	1.16	0.74–1.81
Hoffa fat pad recesses	23	11 (47.8)	1.27	0.50–3.19
Extracapsular synovial cysts^||^	9	2 (22.2)	0.33	0.08–1.35
Popliteus tendon recesses	75	35 (46.7)	0.77	0.49–1.21
Parameniscal cysts (medial)	14	4 (28.6)	0.31	0.10–0.98
Insertional cysts (ACL)	17	12 (70.6)	2.33	0.85–6.39

*Knee complaints during the past 12 mo.

^†^Odds ratio calculated relative to knee complaints (*n* = 70) in the entire study sample. Adjusted for occupation, age, body mass index, knee injuries, and knee-straining sports.

^‡^Confidence interval.

^§^Subgastrocnemius, semimembranosus-gastrocnemius, medial and lateral collateral ligament, biceps femoris, anserine, and semimembranosus-gracilis.

^¶^Prepatellar, superficial and deep infrapatellar.

^||^From capsular defects of the dorsal femoral condyles.

## References

[1] Morrison JL, Kaplan PA (2000). Water on the knee: cysts, bursae, and recesses. *Magnetic Resonance Imaging Clinics of North America*.

[2] McCarthy CL, McNally EG (2004). The MRI appearance of cystic lesions around the knee. *Skeletal Radiology*.

[3] Beaman FD, Peterson JJ (2007). MR imaging of cysts, ganglia, and bursae about the knee. *Magnetic Resonance Imaging Clinics of North America*.

[4] Tschirch FTC, Schmid MR, Pfirrmann CWA, Romero J, Hodler J, Zanetti M (2003). Prevalence and size of meniscal cysts, ganglionic cysts, synovial cysts of the popliteal space, fluid-filled bursae, and other fluid collections in asymptomatic knees on MR imaging. *American Journal of Roentgenology*.

[5] Miller TT, Staron RB, Koenigsberg T, Levin TL, Feldman F (1996). MR imaging of Baker cysts: association with internal derangement, effusion, and degenerative arthropathy. *Radiology*.

[6] Handy JR (2001). Popliteal cysts in adults: a review. *Seminars in Arthritis and Rheumatism*.

[7] Amin S, Goggins J, Niu J (2008). Occupation-related squatting, kneeling, and heavy lifting and the knee joint: a magnetic resonance imaging-based study in men. *Journal of Rheumatology*.

[8] Rytter S, Kirkeskov Jensen L, Bonde JP, Jurik AG, Egund N (2009). Occupational kneeling and meniscal tears: a magnetic resonance imaging study in floor layers. *Journal of Rheumatology*.

[9] Nagura T, Dyrby CO, Alexander EJ, Andriacchi TP (2002). Mechanical loads at the knee joint during deep flexion. *Journal of Orthopaedic Research*.

[10] Thambyah A, Goh JCH, Das De S (2005). Contact stresses in the knee joint in deep flexion. *Medical Engineering and Physics*.

[11] Andriacchi TP (1994). Dynamics of knee malalignment. *Orthopedic Clinics of North America*.

[12] Ward EE, Jacobson JA, Fessell DP, Hayes CW, Van Holsbeeck M (2001). Sonographic detection of Baker’s cysts: comparison with MR imaging. *American Journal of Roentgenology*.

[13] Kivimäki J, Riihimäki H, Hänninen K (1992). Knee disorders in carpet and floor layers and painters. *Scandinavian Journal of Work, Environment and Health*.

[14] Jensen LK, Rytter S, Bonde JP (2010). Exposure assessment of kneeling work activities among floor layers. *Applied Ergonomics*.

[15] Jensen LK, Mikkelsen S, Loft IP, Eenberg W (2000). Work-related knee disorders in floor layers and carpenters. *Journal of Occupational and Environmental Medicine*.

[16] Rytter S, Jensen LK, Bonde JP (2007). Knee complaints and consequences on work status; a 10-year follow-up survey among floor layers and graphic designers. *BMC Musculoskeletal Disorders*.

[17] Marra MD, Crema MD, Chung M (2008). MRI features of cystic lesions around the knee. *Knee*.

[18] Patel SJ, Kaplan PA, Dussault RG, Kahler DM (1998). Anatomy and clinical significance of the horizontal cleft in the infrapatellar fat pad of the knee: MR imaging. *American Journal of Roentgenology*.

[19] Ilahi OA, Younas SA, Labbe MR, Edson SB (2003). Prevalence of ganglion cysts originating from the proximal tibiofibular joint: a magnetic resonance imaging study. *Arthroscopy*.

[20] McLaren DB, Buckwalter KA, Vahey TN (1992). The prevalence and significance of cyst-like changes at the cruciate ligament attachments in the knee. *Skeletal Radiology*.

[21] Peterfy CG, Guermazi A, Zaim S (2004). Whole-organ magnetic resonance imaging score (WORMS) of the knee in osteoarthritis. *Osteoarthritis and Cartilage*.

[22] Rytter S, Egund N, Jensen LK, Bonde JP (2009). Occupational kneeling and radiographic tibiofemoral and patellofemoral osteoarthritis. *Journal of Occupational Medicine and Toxicology*.

[23] Labropoulos N, Shifrin DA, Paxinos O (2004). New insights into the development of popliteal cysts. *British Journal of Surgery*.

[24] Rauschning W (1980). Anatomy and function of the communication between knee joint and popliteal bursae. *Annals of the Rheumatic Diseases*.

[25] Wilson PD, Eyre-Brook AL, Francis JD (1938). A clinical and anatomical study of the semimembranosus bursa in relation to popliteal cyst. *Journal of Bone Joint Surgery*.

[26] Sharrard WJ (1963). Aetiology and pathology of beat knee. *British Journal of Industrial Medicine*.

[27] Thun M, Tanaka S, Smith AB (1987). Morbidity from repetitive knee trauma in carpet and floor layers. *British Journal of Industrial Medicine*.

[28] Myllymäki T, Tikkakoski T, Typpö T, Kivimäki J, Suramo I (1993). Carpet-layer’s knee. An ultrasonographic study. *Acta Radiologica*.

[29] Hayashi D, Roemer FW, Dhina Z (2010). Longitudinal assessment of cyst-like lesions of the knee and their relation to radiographic osteoarthritis and MRI-detected effusion and synovitis in patients with knee pain. *Arthritis Research & Therapy*.

[30] Guermazi A, Hayashi D, Roemer FW (2010). Cyst-like lesions of the knee joint and their relation to incident knee pain and development of radiographic osteoarthritis: the MOST study. *Osteoarthritis and Cartilage*.

[31] Hill CL, Gale DR, Chaisson CE (2003). Periarticular lesions detected on magnetic resonance imaging:
prevalence in knees with and without symptoms. *Arthritis and Rheumatism*.

[32] Dye SF, Vaupel GL, Dye CC (1998). Conscious neurosensory mapping of the internal structures of the human knee without intraarticular anesthesia. *American Journal of Sports Medicine*.

[33] Soifer TB, Levy HJ, Soifer FM, Kleinbart F, Vigorita V, Bryk E (1996). Neurohistology of the subacromial space. *Arthroscopy*.

[34] Pearce N, Checkoway H, Kriebel D (2007). Bias in occupational epidemiology studies. *Occupational and Environmental Medicine*.

